# Preventive Effects of *Eclipta prostrata* and *Hordeum vulgare* Extract Complex on Precocious Puberty in Danazol- and High-Fat Diet-Induced Rat Models

**DOI:** 10.3390/ijms262211158

**Published:** 2025-11-18

**Authors:** Young-Sik Kim, Taekil Eom, Yongbin Kim, Jinhui Rhee, Hongjun Kim

**Affiliations:** 1 Department of Herbology, College of Korean Medicine, Woosuk University, Jeonju 55338, Republic of Korea; yjbsik@woosuk.ac.kr (Y.-S.K.); taekil7@hanmail.net (T.E.); drag8070@hanmail.net (Y.K.); qa6411@gmail.com (J.R.); 2 Department of Formula Science, College of Korean Medicine, Woosuk University, Jeonju 55338, Republic of Korea

**Keywords:** precocious puberty, *Eclipta prostrata*, *Hordeum vulgare*, GnRH, danazol, high fat diet

## Abstract

Precocious puberty, characterized by the abnormally early onset of secondary sexual development, has been increasing in prevalence worldwide. Current pharmacological treatments, including GnRH agonists, are effective but associated with adverse effects, highlighting the need for safer alternatives. In this study, we investigated the preventive effects of an herbal extract complex composed of *Eclipta prostrata* and *Hordeum vulgare* (EHEC) on precocious puberty induced by danazol administration and a high-fat diet (HFD) in rat models. EHEC delayed vaginal opening (VO) and reduced ovarian maturation in both models. Furthermore, EHEC attenuated the elevation in hypothalamic GnRH mRNA expression observed in both models, without affecting body weight. These findings suggest that EHEC modulates the hypothalamic–pituitary–gonadal axis and may serve as a potential natural therapeutic agent for the prevention of precocious puberty.

## 1. Introduction

Puberty is a critical developmental stage regulated by the hypothalamic–pituitary–gonadal (HPG) axis [1,2,3]. It begins with the pulsatile release of gonadotropin-releasing hormone (GnRH) from the hypothalamus, which in turn stimulates the pituitary gland to release luteinizing hormone (LH) and follicle-stimulating hormone (FSH) [4,5,6]. These gonadotropins subsequently induce estrogen and testosterone production in the gonads, thereby promoting the development of secondary sexual maturation [7,8].

Precocious puberty refers to the premature activation of the hypothalamic–pituitary–gonadal (HPG) axis, defined as the onset of secondary sexual characteristics before 8 years of age in girls and 9 years in boys [9]. This sex difference reflects the fact that physiological puberty normally begins earlier in girls due to earlier hypothalamic maturation and greater gonadotropin sensitivity compared to boys [10,11]. The etiology of precocious puberty is heterogeneous, encompassing idiopathic, genetic, neurological, and environmental factors [12]. Among these environmental factors, the increasing prevalence of childhood obesity has been identified as a significant contributor to the earlier onset of puberty, particularly in girls [13,14]. Precocious puberty can be classified as either central precocious puberty (CPP), which is GnRH-dependent, or peripheral precocious puberty (PPP), which is independent of hypothalamic GnRH secretion [15,16,17]. CPP results from the premature onset of pulsatile GnRH secretion, which subsequently stimulates increased LH and FSH release, leading to early activation of the HPG axis [9,18]. In contrast, PPP can result from conditions such as hypothyroidism, hormone-secreting tumors, excess androgens, and ovarian cysts [9,15].

The sequelae of precocious puberty are well-established and include premature epiphyseal closure, reduced final adult stature, increased risk of hormone-related malignancies, and psychological challenges such as anxiety, behavioral disturbances, and stress-related disorders [19,20,21]. Although GnRH agonist therapy remains clinically effective, the rising global incidence of precocious puberty and the known adverse effects associated with this treatment underscore the need to explore alternative therapeutic strategies [22,23]. In many Asian countries, herbal medicines have a long history of use for the management of precocious puberty and remain relevant in current clinical practice [24,25]. While most contemporary research has focused on pharmacological molecules such as GnRH agonists, growing interest in natural products, including herbal medicines, underscores the potential for novel management strategies [26]. Although recent years have seen increased attention to the study of herbal medicine in the treatment of precocious puberty [27,28,29,30,31,32], the evidence base remains limited.

*Eclipta prostrata* (L.) L. (commonly known as false daisy, Hanlyeoncho in Korean medicine), a member of the Asteraceae family, has been traditionally used in East Asian traditional herbal medicine for hepatorenal support, blood cooling, and hemostasis [33]. Pharmacological studies have demonstrated hepatoprotective, anti-inflammatory, analgesic, anticancer, and neuroprotective activities [34,35]. Malt (*Hordeum vulgare* L.), derived from sprouted barley, has been used in traditional medicine for the management of gastrointestinal disturbances, including indigestion and anorexia [36,37]. Furthermore, contemporary pharmacological studies have revealed that malt possesses antioxidant, skin-whitening, cardioprotective, anti-allergic, and immunomodulatory effects [38,39].

Recent studies suggest that herbal combinations, including *E. prostrata* and *H. vulgare*, may exert regulatory effects on the endocrine system [40,41]. However, their role in preventing precocious puberty has not yet been elucidated. Therefore, this study aimed to investigate the effects of an EHEC on precocious puberty in danazol- and HFD-induced rat models, with a focus on modulation of the HPG axis.

## 2. Results

### 2.1. Quantification of Chlorogenic Acid and Wedelolactone in EHEC

**Figure 1:**
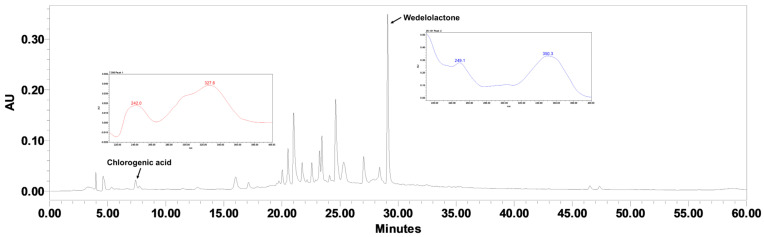
High−performance liquid chromatogram of EHEC at 351 nm. Peaks corresponding to chlorogenic acid and wedelolactone are labeled, confirming their presence in the extract.

The representative HPLC chromatogram of the EHEC is shown in Figure 1, with the marker compounds chlorogenic acid and wedelolactone labeled on the chromatogram. The presence of these compounds in the extract was confirmed by comparison with reference standards. The contents of chlorogenic acid and wedelolactone in the EHEC were quantified as 0.58 mg/g and 3.79 mg/g, respectively.

### 2.2. Effects of EHEC on Vaginal Opening Delay in Danazol-Induced Precocious Puberty Rats

**Figure 2:**
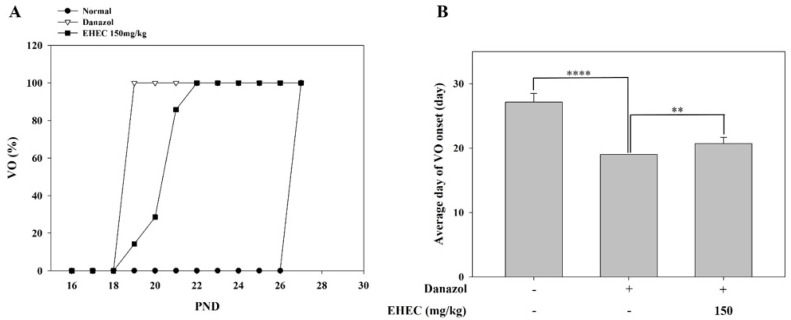
Effects of EHEC on vaginal opening (VO) in a danazol-induced precocious puberty model. (**A**) Daily cumulative percentages of VO were plotted for the normal (*n* = 7), control (*n* = 9), and EHEC-150 groups (*n* = 7). No statistical analysis was performed on the daily cumulative data. (**B**) The average day of VO onset is shown for each group, with statistical comparisons conducted among the groups. Statistical significance was determined via one-way ANOVA, and differences are indicated by ** *p* < 0.01, **** *p* < 0.0001.

Danazol administration on postnatal day (PND) 5 induced precocious puberty in female rats. In the normal group, VO occurred at approximately PND 27, whereas in the danazol-treated group (control), it occurred on approximately PND 19, about eight days earlier. Treatment with EHEC delayed VO to around PND 21, about two days later than the control group (Figure 2A,B). These results indicate that EHEC alleviates danazol-induced precocious puberty in female rats by delaying VO.

### 2.3. Effects of EHEC on Body, Uterine, and Ovarian Weights in a Danazol-Induced Precocious Puberty Model

**Figure 3:**
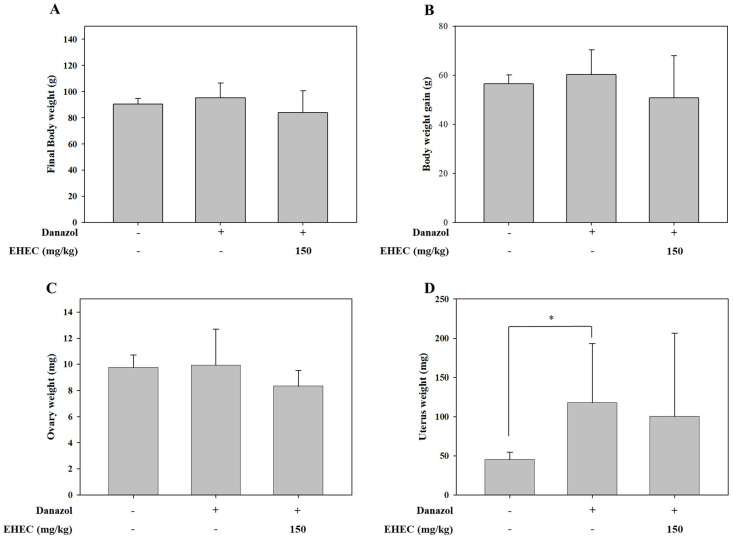
Effects of EHEC treatment on body weight, ovary weight, and uterine weight in danazol-induced precocious puberty models. (**A**) Final body weight, (**B**) body weight gain, (**C**) ovary weight, and (**D**) uterine weight were measured in EHEC-150 group compared with the control group. Data are presented as the mean ± SD (Normal: *n* = 7, Control: *n* = 9, EHEC-150: *n* = 7). Statistical significance is indicated by * *p* < 0.05.

To further evaluate treatment effects on reproductive development, body weight, body weight gain, uterine weight, and ovarian weight were measured. Body weight, body weight gain, and ovary weight did not differ significantly among groups (Figure 3A–C). However, uterine weight was significantly increased in the control group compared with the normal group. EHEC treatment partially reduced uterine weight, although the difference did not reach statistical significance (Figure 3D).

### 2.4. Influence of EHEC on Ovarian Follicle Formation in the Danazol-Induced Precocious Puberty Model

**Figure 4:**
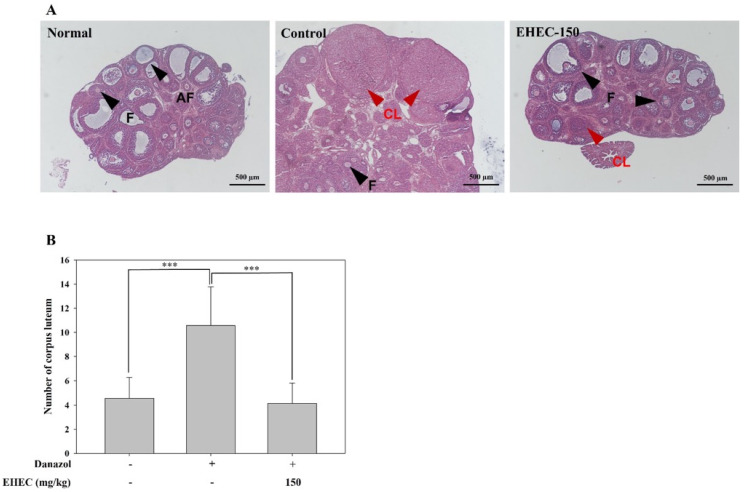
Histological analysis of ovarian tissues and quantification of corpora lutea in danazol-induced precocious puberty models treated with EHEC. (**A**) Representative H&E-stained ovarian tissue sections from normal, control, and EHEC-150 groups. Key structures indicated are follicles (F), atretic follicles (AF), and corpora lutea (CL). (**B**) Quantification of the number of corpora lutea in each group. Data are presented as the mean ± SD (*n* = 7/group). Statistical significance is indicated by *** *p* < 0.001.

In this analysis, uterine and ovarian tissues were examined to identify potential morphological differences. Histological examination of the ovaries using hematoxylin and eosin (H&E) staining revealed that the control group exhibited overdeveloped corpora lutea (Figure 4A). Notably, the ovaries of rats treated with EHEC showed histological characteristics comparable to those of the normal group, suggesting that EHEC may alleviate the onset of danazol-induced precocious puberty and restore ovarian histological characteristics to normal levels (Figure 4B).

### 2.5. Effects of EHEC on GnRH and GnRHR Gene Expression in the Danazol-Induced Precocious Puberty Model

**Figure 5:**
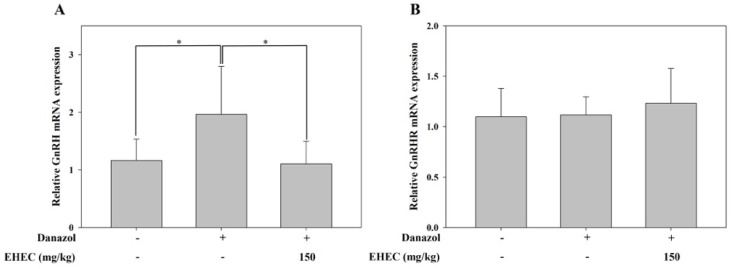
Effects of EHEC on hypothalamic GnRH and pituitary GnRHR mRNA expression in danazol-induced precocious puberty models. (**A**) Relative expression of hypothalamic GnRH mRNA and (**B**) relative expression of pituitary GnRHR mRNA were measured in normal, control, and EHEC-150 groups. Data are presented as the mean ± SD (*n* = 6/group). Statistical significance is indicated by * *p* < 0.05.

To determine whether the induction of precocious puberty was associated with GnRH, a reproductive regulatory hormone produced in the hypothalamus, we analyzed the expression levels of GnRH and its receptor, GnRHR. RT-qPCR analysis revealed that hypothalamic GnRH expression was significantly increased in the control group compared to the normal group (Figure 5A). Treatment with EHEC significantly attenuated this increase. In contrast, no significant differences in pituitary GnRHR expression were observed among the groups (Figure 5B).

### 2.6. Effect of EHEC on Delaying Vaginal Opening in Rats with High-Fat Diet-Induced Precocious Puberty

**Figure 6:**
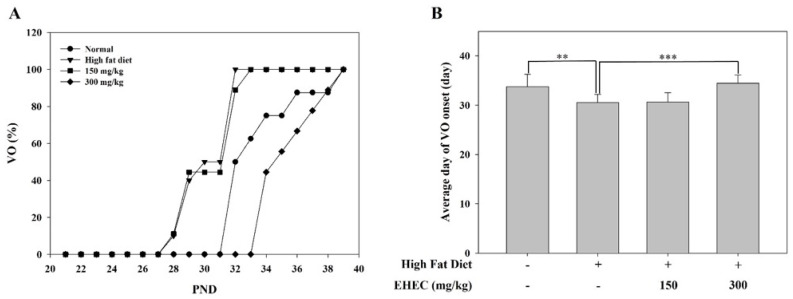
Effects of EHEC on vaginal opening (VO) in HFD-induced precocious puberty model. (**A**) Daily cumulative percentages of VO by PND are plotted for four groups: normal group (*n* = 8), HFD group (*n* = 10), EHEC-150 group (*n* = 9), and EHEC-300 group (*n* = 10). No statistical analysis was performed on the daily cumulative data in panel (**A**). (**B**) The mean VO onset day is presented for each group. Statistical significance among groups was evaluated by one-way ANOVA with post hoc test. Data are presented as the mean ± SD, and significant differences are denoted as ** *p* < 0.01, *** *p* < 0.001.

To determine whether EHEC suppresses obesity-related precocious puberty, rats were fed a HFD starting at PND 21, and VO was monitored following the administration of either the HFD (HFD group) or a normal diet (Normal group) (Figure 6A). In the control group, VO was first observed on PND 28, and by PND 31, all animals had exhibited VO. In the EHEC-150 group, VO was also first detected on PND 28, with complete VO in all animals occurring later, on PND 33. In the EHEC-300 group, VO was first observed on PND 34, and all animals exhibited VO by PND 39. Compared with the control group, 300 mg/kg EHEC administration significantly delayed the onset of VO (Figure 6B).

### 2.7. Effects of EHEC on Body, Uterine, and Ovarian Weights in a HFD-Induced Precocious Puberty Model

**Figure 7:**
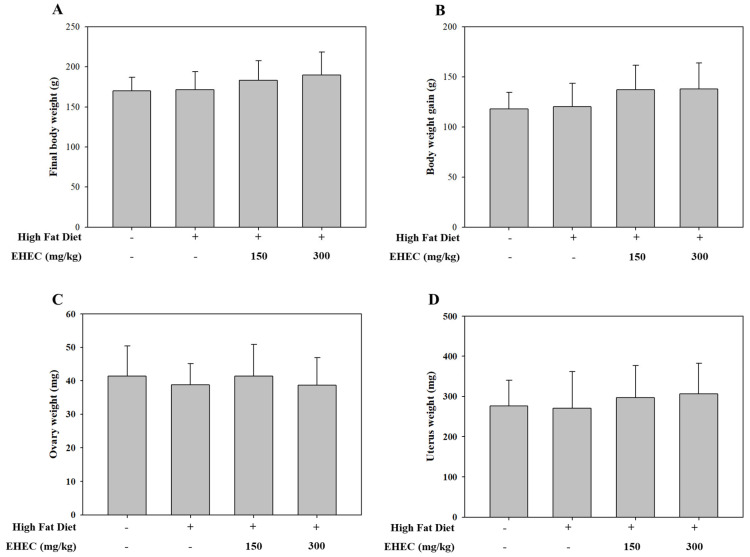
Effects of EHCE on body, ovary, and uterus weight in the HFD-induced precocious puberty model. (**A**) Final body weight (g), (**B**) body weight gain (g), (**C**) ovary weight (mg), and (**D**) uterine weight (mg) are shown for each group: Normal, Control, EHEC-150 and EHEC-300 groups. Data are expressed as the mean ± SD (*n* = 8 per group).

In addition to the delayed VO observed, body weight gain and reproductive organ weights were evaluated to assess the effects of EHEC on reproductive development in the HFD-induced precocious puberty model. Rats were fed an HFD to induce precocious puberty and were subsequently administered EHEC at either a low dose (150 mg/10 mL/kg) or a high dose (300 mg/10 mL/kg). As shown in Figure 7A,B, there were no statistically significant differences in final body weight or body weight gain between the control and EHEC-treated groups. Similarly, ovarian (Figure 7C) and uterine weights (Figure 7D) did not differ significantly among the experimental groups. These results indicate that EHEC administration did not affect the general growth parameters or reproductive organ weights under HFD-induced conditions.

### 2.8. Influence of EHEC on Ovarian Follicle Formation in the HFD-Induced Precocious Puberty Model

**Figure 8:**
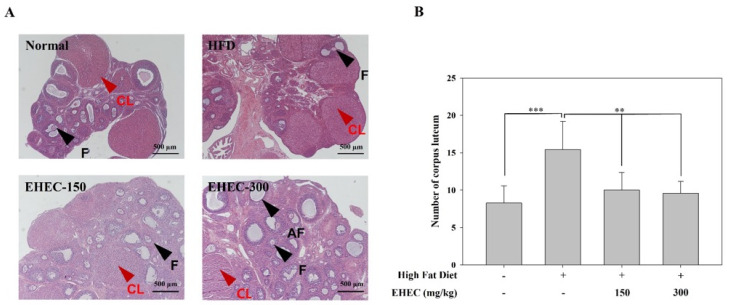
Histological analysis of ovarian tissues in the HFD-induced precocious puberty model. (**A**) Representative images of H&E-stained ovarian sections from each group: normal, control, EHEC-150 and EHEC-300, indicating corpora lutea (CL), atretic follicles (AF), and follicles (F). Scale bar = 500 µm. (**B**) Quantification of corpora lutea number in each group. Data are presented as the mean ± SD (*n* = 7 per group). Statistical analysis was performed using one-way ANOVA with post hoc test; ** *p* < 0.01, *** *p* < 0.001 compared with the HFD group.

Histological analysis of ovaries was performed using hematoxylin-eosin (H&E) staining (Figure 8A). In the HFD group, the number of corpora lutea were more than twofold, but the number of follicles remained unchanged compared to the normal group. The ovaries of the EHEC-treated group exhibited histological characteristics similar to those of the normal group. EHEC administration at 150 and 300 mg/kg significantly reduced the number of corpora lutea, restoring it to levels similar to those in the normal group. Despite EHEC administration, the number of follicles remained unchanged. This suggests that EHEC can alleviate the onset of HFD-induced precocious puberty and restore ovarian histological characteristics to normal levels (Figure 8B).

### 2.9. Influence of EHEC on GnRH and GnRHR Gene Expression in the HF Diet Model

**Figure 9:**
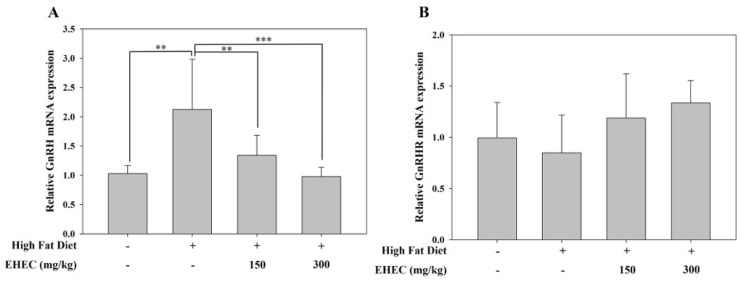
Effects of EHEC on hypothalamic GnRH and pituitary GnRHR mRNA expression in the HFD-induced precocious puberty model. (**A**) Relative GnRH mRNA expression levels in the hypothalamus for each group: normal, control, EHEC-150 and EHEC-300. (**B**) Relative GnRHR mRNA expression levels in the pituitary. All mRNA levels were quantified by RT-qPCR using the 2^−ΔΔCt^ method. Data are presented as the mean ± SD (*n* = 6 per group). Statistical significance was determined by one-way ANOVA with the post hoc test; ** *p* < 0.01, *** *p* < 0.001 compared with the HFD group.

Similar to the danazol-induced precocious puberty model, we investigated the role of the reproductive hormone GnRH in HFD-induced precocious puberty. Hypothalamic GnRH expression was significantly increased in the control group compared to the normal group (Figure 9A). EHEC administration suppressed GnRH expression. Similar to the danazol model, pituitary GnRHR expression did not differ significantly among any of the HFD-fed groups (Figure 9B).

## 3. Discussion

Precocious puberty is defined as the development of secondary sexual characteristics at an earlier age than is considered normal in children [42]. Recent decades have shown a secular trend toward earlier pubertal onset worldwide, with meta-analytic data indicating that the mean age at thelarche has advanced by approximately 0.24 years per decade in girls [43]. Consistent with this global pattern, national registry data from Korea demonstrate a marked increase in the incidence and prevalence of CPP since the early 2000s [44]. Recently, various herbal medicine formulations have been reported to alleviate symptoms associated with precocious puberty [24,45]. The present study demonstrates that EHEC exerts preventive effects in both danazol- and HFD-induced rat models of precocious puberty. EHEC delayed vaginal opening, reduced ovarian maturation, and suppressed hypothalamic GnRH expression. These findings indicate that EHEC regulates the hypothalamic–pituitary–gonadal (HPG) axis, thereby mitigating early onset of puberty.

Danazol is a synthetic derivative of 17α-ethinyl testosterone that induces precocious puberty in neonatal rats by disrupting the HPG axis. It primarily suppresses the pituitary secretion of gonadotropins (LH and FSH), which alters hormonal feedback and leads to premature activation of reproductive development [46,47]. In our study, the danazol-treated rats exhibited features consistent with those previously reported for this well-established experimental model, including earlier VO, increased uterine weight, and enhanced corpus luteum formation [28,48]. Administration of EHEC attenuated these danazol-induced effects, indicating that EHEC suppresses premature ovarian maturation and pubertal onset. Recently, an herbal formulation composed of Anemarrhenae rhizome and Phellodendri cortex extracts (1:1) has been reported to delay VO and attenuate uterine maturation in a danazol-induced precocious puberty rat model. This combination, administered at 200 mg/kg, produced approximately a three-day delay in pubertal onset without markedly affecting ovarian or uterine histology [49]. Similarly, administration of a complex of 12 herbal medicines, including Coicis semen—a Poaceae family plant taxonomically related to *H. vulgare* used in the present study—delayed VO and suppressed uterine maturation in the same model [24]. These findings collectively indicate that certain herbal mixtures may modulate the hypothalamic–pituitary–gonadal axis and delay pubertal progression under experimental conditions, mimicking precocious puberty.

Childhood obesity has been increasingly recognized as a significant contributing factor to earlier pubertal onset rather than a direct primary cause. Epidemiological studies have reported that overweight and obese children—particularly girls—have a higher risk of earlier puberty compared with their normal-weight peers (odds ratio ≈ 2.2 in pooled analyses) [13,14]. Excess adiposity promotes adipocyte maturation and the release of adipokines such as leptin, which may activate hypothalamic kisspeptin neurons and enhance Kiss-1 and GnRH secretion, thereby stimulating LH and FSH release and accelerating sexual maturation [50,51,52]. In agreement with these observations, animal studies have demonstrated that rats fed a HFD show earlier VO and accelerated uterine and ovarian maturation compared to controls, providing a reproducible model of obesity-related precocious puberty [53]. In the present study, we observed that VO and ovarian maturation occurred earlier in the control group, whereas EHEC administration normalized these parameters to levels comparable to those in the normal group. These findings suggest that EHEC may ameliorate or delay obesity-related pubertal advancement in this animal model.

GnRH is recognized as a key regulator of the HPG axis during sexual maturation in adolescence. GnRH secreted from hypothalamic neurons binds to GnRHR in the pituitary gland, thereby increasing the secretion of LH and FSH [4,5,6]. In this study, both danazol and a HFD increased hypothalamic GnRH mRNA expression, whereas EHEC administration attenuated this effect. However, no significant changes were observed in GnRHR expression in the pituitary. These findings suggest that EHEC may modulate the hypothalamic component of the HPG axis by downregulating GnRH gene expression, although further studies are required to confirm whether this translates to altered GnRH secretion. Clinically, GnRH analogs such as leuprolide are the standard therapy for CPP, acting by suppressing hypothalamic GnRH signaling [10]. In this context, EHEC could serve as a potential natural adjunct that modulates the same pathway with potentially fewer adverse effects than conventional pharmacologic therapy. Various herbal medicines have been investigated for preventing precocious puberty by modulating hypothalamic GnRH expression. For example, the traditional Chinese prescription Fuyou formula, which notably contains *H. vulgare*, was reported to suppress GnRH expression in both GT1-7 hypothalamic GnRH-secreting neuronal cells and in a danazol-induced precocious puberty model [54]. This overlap is particularly relevant, as H. vulgare is also one of the principal components used in the present study. Similarly, the Estrogen Inhibition Formula, composed of 12 medicinal plants with a distinct composition from that employed here, has been shown to suppress hypothalamic GnRH expression in the same experimental model [28]. In obesity-induced precocious puberty, epigallocatechin gallate derived from green tea was found to suppress GnRH expression and thereby ameliorate precocious puberty [55]. Consistent with these findings, EHEC treatment in the present study downregulated hypothalamic GnRH mRNA expression in both the danazol- and HFD-induced models, suggesting that modulation of hypothalamic GnRH signaling may be a common mechanism underlying the anti-pubertal effects of certain herbal formulations. Furthermore, during normal puberty, GnRHR expression and responsiveness in the pituitary are transiently enhanced in response to GnRH stimulation and subsequently stabilize [56]. Although these dynamics were not directly evaluated here, previous evidence suggests that early increases in GnRH pulse frequency can drive pubertal progression, even in the absence of major changes in GnRHR expression [57]. This may explain why EHEC modulated hypothalamic GnRH expression without significantly altering the pituitary GnRHR levels.

We also used H&E staining to quantify the number of corpora lutea in the ovaries. An increase was observed in both the danazol- and the HFD-induced precocious puberty groups, whereas EHEC administration reduced the number of corpora lutea. To test whether these changes in ovarian follicles and corpora lutea were associated with alterations in gonadotropins, we measured the serum LH and FSH levels. However, no significant differences were observed among the experimental groups. LH and FSH, secreted from the pituitary gland, regulate corpora lutea formation by acting on the ovary to induce ovarian maturation [58]. Previous studies investigating natural compounds such as luteolin and hydroxytyrosol have shown that their consumption suppresses corpora lutea formation associated with precocious puberty [48,59]. In this study, luteinization was reduced without corresponding decreases in circulating LH level, suggesting a direct ovarian effect, possibly through the suppression of luteinization or downregulation of ovarian LH receptor expression [60]. These effects may arise from altered ovarian steroidogenesis, including estradiol and progesterone synthesis [61]. Additionally, because LH is secreted in a pulsatile manner, single time-point measurements may have obscured subtle changes [51]. Taken together, these findings suggest that luteal regression may occur independently of systemic LH levels, underscoring the importance of evaluating both central and peripheral mechanisms in models of precocious puberty. Importantly, our results demonstrated that EHEC administration reduced corpora lutea formation, supporting its potential role in delaying pubertal onset and preventing precocious puberty.

The identification and quantification of bioactive compounds in EHEC were performed using HPLC analysis. Among the analytes screened, wedelolactone and chlorogenic acid (CGA) were detected and quantified, whereas other compounds previously reported in *E. prostrata* and *H. vulgare* were not identified under the analytical conditions employed. Wedelolactone, a coumestan compound prevalent in Asteraceae plants, is known for its antioxidant, anti-inflammatory, antiviral, and hepatoprotective activities. [62]. Previous studies have shown that wedelolactone exhibits phytoestrogen-like effects and reduces LH, FSH, and estradiol levels in postmenopausal osteoporosis models [63]. Although the postmenopausal endocrine state differs from that of precocious puberty, the observed downregulation of gonadotropic hormones suggests that wedelolactone may exert suppressive effects on the hypothalamic–pituitary–gonadal axis, potentially contributing to delayed pubertal onset.

CGA, a phenolic compound composed of caffeic and quinic acids, also exhibits antioxidant and anti-inflammatory properties [64], but it was not among the most abundant components in EHEC (Figure 1). In polycystic ovary syndrome models, CGA has been shown to decrease LH and increase FSH levels, indicating possible relevance to gonadotropic regulation [65,66]. While both wedelolactone and CGA possess broad biological activities that may influence endocrine function, the present findings do not establish a causal link between these constituents and EHEC’s anti-pubertal effects. Further studies are warranted to identify the active compounds and elucidate the molecular mechanisms underlying these effects.

Our study demonstrates that EHEC effectively attenuates precocious puberty in two distinct animal models: a danazol-induced model, representing idiopathic/central precocious puberty, and a HFD-induced model, reflecting obesity-associated precocious puberty. These results suggest the broad therapeutic potential of EHEC. However, the translational relevance of these findings requires further validation in human studies. Future research should focus on elucidating the molecular targets and mechanisms of action of EHEC, as well as identifying its bioactive components. In addition, investigations into pharmacokinetics, optimal dosing, and safety profile of EHEC are essential to support its potential clinical application as a complementary intervention for both idiopathic and obesity-related precocious puberty.

## 4. Materials and Methods

### 4.1. Preparation of E. prostrata and H. vulgare Mixture

The aerial parts of *E. prostrata* and the grains of *H. vulgare* were purchased in dried form from a commercial supplier (Naemomme Dah, Ulsan, Republic of Korea) and identified by Prof. Hongjun Kim. Each plant material was individually pulverized and extracted using a reflux apparatus with 10 volumes of 30% ethanol at 85 °C for 2 h. The extract was filtered through Whatman filter paper, concentrated under reduced pressure using a rotary evaporator (Eyela, Tokyo, Japan), and freeze-dried using a freeze dryer (Ilshin Biobase, Dongducheon, Republic of Korea) to yield powdered extracts, with extraction yields of 17.4% for *E. prostrata* and 6.9% for *H. vulgare*. Each extract was stored as a powder at 4 °C and weighed separately before use. For experimental administration, the two extracts were combined in a 1:1 ratio to prepare the mixed extract (EHEC).

### 4.2. High-Performance Liquid Chromatography (HPLC) Analysis

The contents of wedelolactone and chlorogenic acid in the EHEC were measured using a Waters Alliance HPLC system equipped with a 2695 separations module (Waters Co., Milford, CT, USA) coupled with a 2998 photodiode array detector. Compounds in the EHEC were separated on a YMC-Pack ODS A column (250 mm × 4.6 mm i.d., 5 μm particle size; YMC Co. Ltd., Kyoto, Japan), and the column temperature was maintained at 30 °C. The mobile phase consisted of 0.1% (*v*/*v*) phosphoric acid in distilled water (A) and 0.1% (*v*/*v*) phosphoric acid in acetonitrile (B) at a flow rate of 0.8 mL/min. The gradient elution program was as follows: 15% B held from 0 to 10 min, linearly increased from 15% to 70% B from 10 to 45 min, from 70% to 90% B from 45 to 50 min, held at 90% B from 50 to 53 min, and decreased from 90% to 15% B from 53 to 60 min. The injection volume was 10 μL. Detection wavelengths were set at 351 nm for wedelolactone from *E. prostrata* and 325 nm for chlorogenic acid from *H. vulgare*. Standards were sourced from Biofron (La Mirada, CA, USA, wedelolactone; Cat. No BF-W3002) and Tauto BioTech (Shanghai, China, CGA; E-0075). Calibration curves showed good linearity for both analytes: wedelolactone, y = 26,905x + 661,663 (R^2^ = 0.9957); CGA, y = 29,569x − 19,020 (R^2^ = 0.9998), where y is peak area and x is concentration.

### 4.3. Animals, Housing, and Ethical Approval

All experiments were conducted using female Sprague–Dawley (SD) rats obtained from Samtako Bio Korea Co. Ltd. (Osan, Republic of Korea). Animals were housed in polycarbonate cages (JD-C-02, Jeungdo B&P, Seoul, Republic of Korea; 260 mm × 420 mm × 180 mm, W × D × H) equipped with stainless-steel wire lids under controlled temperature conditions (23 ± 2 °C) with a 12 h light/dark cycle. Animals had ad libitum access to food and water. All animal procedures were performed in accordance with the ARRIVE guidelines (https://arriveguidelines.org, accessed on 1 September 2025) and were approved by the Institutional Animal Care and Use Committee (IACUC) of Woosuk University. For the danazol-induced precocious puberty model, approval was obtained under Approval No. 2022-07. For the high-fat diet-induced precocious puberty model, approval was obtained under Approval No. 2025-01.

### 4.4. Danazol-Induced Precocious Puberty Model Design

On postnatal day 1 (PND 1), female SD rats and their dams aged 11–13 weeks were obtained and acclimated for three days before the start of the experiment. The study included three groups: normal (*n* = 7), control (*n* = 9), and EHEC-150 treated groups (*n* = 7). On PND 5, a single subcutaneous injection of danazol (C3644, ≥99% purity; 300 µg/30 µL; Apexbio, Houston, TX, USA) dissolved in ethylene glycol:ethanol (1:1, *v*/*v*) was administered to all groups except the normal group, following the methodology described by *Bai* et al. [27]. The EHEC group received daily intragastric doses of the combined extract of *E. prostrata* and *H. vulgare* (1:1 ratio, 150 mg/kg), suspended in distilled water, starting on PND 15 (Supplementary Figure S1A). Both the normal and control groups received an equivalent volume of distilled water. Body weight was recorded daily throughout the experimental period.

#### Monitoring Vaginal Opening and Estrous Cycle in Danazol-Induced Precocious Puberty Model

The vaginal opening (VO) was inspected daily by visually examining the vaginal orifice. The estrous cycle was monitored daily starting from PND 15, following the initiation of danazol administration. Vaginal smears were prepared by flushing with physiological saline and examined microscopically. The appearance of VO was considered indicative of puberty onset.

### 4.5. High-Fat Diet-Induced Precocious Puberty Model Design

On PND 20, female SD rats were randomly assigned to four groups: normal (*n* = 8), control (*n* = 10), EHEC-150 (150 mg/kg, *n* = 9) and EHEC-300 (300 mg/kg, *n* = 10) groups. Pups were separated from their dams, and body weight was recorded. The combined extract of *E. prostrata* and *H. vulgare* (1:1 ratio) was suspended in distilled water before administration. The normal and control groups received distilled water only. The extract was administered by daily oral gavage starting on PND 20 (Supplementary Figure S1B). Body weight and food intake were measured daily throughout the experimental period. To establish the HFD-induced precocious puberty model, rats were fed a HFD (D12492, Research Diets, Inc., New Brunswick, NJ, USA; 60% kcal from fat), while normal groups received a control diet (D12450B, Research Diets, Inc.) containing 10% kcal from fat, with matched sucrose content. All diets were provided ad libitum.

#### Monitoring Vaginal Opening and Estrous Cycle in HFD-Induced Precocious Puberty Model

The vaginal opening (VO) was inspected daily by visually examining the vaginal orifice. The estrous cycle was monitored daily starting from PND 21, after separation from the dams. Vaginal epithelial cells were stained with eosin Y to confirm the presence of cornified cells. The appearance of VO was considered indicative of puberty onset.

### 4.6. Euthanasia Procedure

Following the detection of vaginal opening and identification of the second diestrus phase, rats were euthanized under isoflurane anesthesia (80 cc/min, Hana PH, Seoul, Republic of Korea) via decapitation, in accordance with the AVMA (*American Veterinary Medical Association*) Guidelines for the Euthanasia of Animals (2020 Edition). The uterus and ovaries were immediately collected and weighed for analysis.

### 4.7. Organ Collection and Analysis

Following euthanasia at the second diestrus phase, the uterus and ovaries were collected and weighed. Immediately following decapitation, trunk blood was also collected to obtain serum for hormonal analysis. The hypothalamus and pituitary gland tissues were dissected and collected from all groups in both experimental models for PCR analysis.

### 4.8. Histological Preparation and Staining of Ovarian Tissue

Ovarian tissues were fixed in formalin for subsequent hematoxylin (H3136, Sigma-Aldrich Co., St. Louis, MO, USA) and eosin (T0035, TCI, Tokyo, Japan) (H&E) staining to examine tissue morphology. Fixed samples were embedded, sectioned at 5 µm thickness, and stained with H&E. Briefly, sections were dewaxed in xylene and rehydrated through a graded ethanol series (100%, 90%, 80%, and 70%). The sections were then stained with hematoxylin for 5 min, rinsed in water, and counterstained with eosin for 1 min. After staining, the sections were dehydrated through ascending ethanol concentrations (70%, 80%, 90%, and 100%) and cleared in xylene three times.

### 4.9. RNA Extraction and mRNA Expression Analysis

To measure GnRH and GnRHR expression, rats were euthanized after anesthesia, and the hypothalamus and pituitary tissues were collected. Total RNA was then extracted from these tissues using RNAiso Plus (Takara, 9108; Kusatsu, Japan) following the manufacturer’s instructions. For cDNA synthesis, 500 ng of total RNA was used per reaction with the PrimeScript RT Reagent Kit (Takara, RR036A; Kusatsu, Japan). The resulting cDNA was diluted to an optimal concentration for subsequent real-time PCR analysis. Quantitative real-time PCR was performed using the SYBR Premix Ex Taq Kit (Takara, RR430; Kusatsu, Japan) following the manufacturer’s protocol. The primer sequences used for rat tissue were as follows: GAPDH (Forward) 5′-GGCACAGTCAAGGCTGAGAATG-3′, GAPDH (Reverse) 5′-ATGGTGGTGAAGACGCCAGTA-3′; GnRH (Forward) 5′-CTACTGCTGACTGTGTGTTTG-3′, GnRH (Reverse) 5′-CATCTTCTTCTGCCTGGCTTC-3′; GnRHR (Forward) 5′-GATGTCTGCAGCCTGAGTCCC-3′, GnRHR (Reverse) 5′-AGGCATTAACGAGTTCCTGGG-3′.

### 4.10. Statistical Analysis

Statistical analyses were performed using Prism 8 (GraphPad Software, Inc., San Diego, CA, USA). Data were analyzed via unpaired the Student’s *t*-test or one-way ANOVA followed by Dunnett’s multiple comparisons test, when the normality of distribution and homogeneity of variances were confirmed using Bartlett’s test. For non-normally distributed data, the Kruskal–Wallis test followed by Dunn’s post hoc test was applied. In cases of heterogeneity of variances, Brown–Forsythe ANOVA with Dunnett’s T3 multiple comparisons test was used. All data are presented as the mean ± standard deviations (SD). A *p* value < 0.05 was considered statistically significant. Significance levels were denoted as * *p* < 0.05, ** *p* < 0.01, *** *p* < 0.001, **** *p* < 0.0001, and ns (not significant).

## 5. Conclusions

This study demonstrated that an EHEC alleviated the manifestations of precocious puberty in danazol- and HFD-induced rat models. EHEC delayed pubertal onset, reduced ovarian maturation, and suppressed hypothalamic GnRH expression, suggesting a modulatory effect on the HPG axis. These findings indicate that EHEC may serve as an effective natural alternative for the prevention of precocious puberty. Rather than as a stand-alone preventive therapy, EHEC may be considered a potential adjunctive supplement to conventional GnRH analog treatment. Further mechanistic investigations and clinical studies are warranted to validate its safety and therapeutic relevance in humans.

## Data Availability

The original contributions presented in this study are included in the article/Supplementary Material. Further inquiries can be directed to the corresponding author.

## Supplementary Materials

The following supporting information can be downloaded at: https://www.mdpi.com/article/10.3390/ijms262211158/s1.
