# Supplementary material for: Preventive Effects of Eclipta prostrata and Hordeum vulgare Extract Complex on Precocious Puberty in Danazol- and High-Fat Diet-Induced Rat Models
Source: Int J Mol Sci. 2025 Nov 18;26(22):11158. doi: 10.3390/ijms262211158 (PMC12652678; doi:10.3390/ijms262211158)
Supplement: Supplementary file 1 [file ijms-26-11158-s001.zip › ijms-3945503-supplementary.pdf]

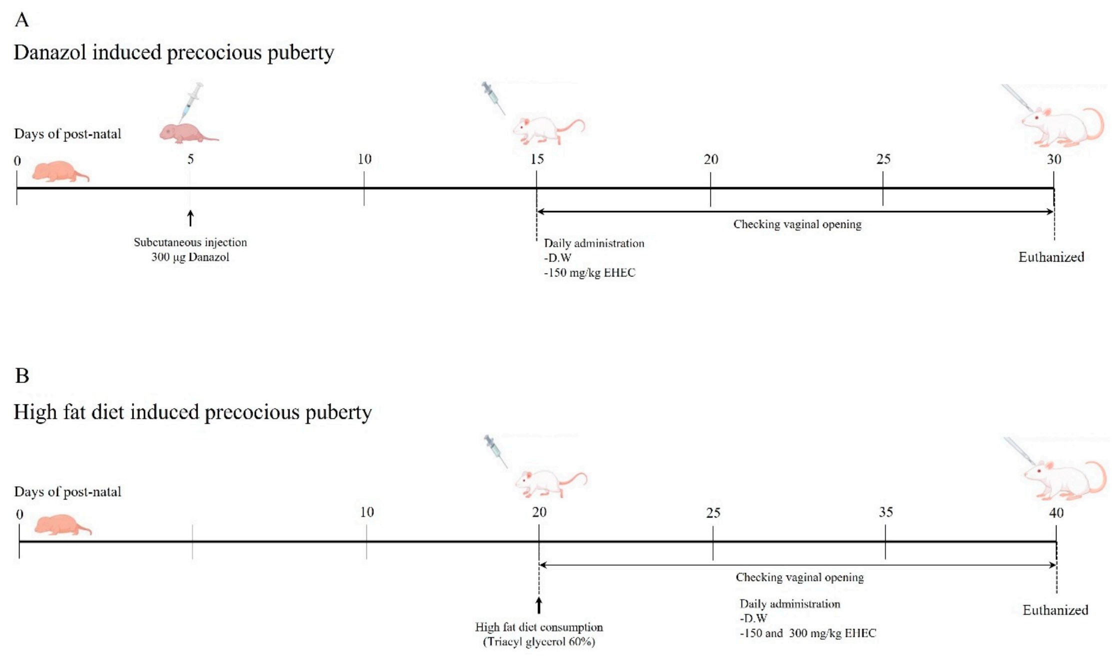

Figure S1. Flowchart outlining the *in vivo* experimental design and assay for this study. (**A**) Schematic representation of the Danazol induced precocious puberty experimental design. (**B**) Schematic representation of the High fat diet induced precocious puberty experimental design.
